# Community transmission of SARS-CoV-2 during the Delta wave in New York City

**DOI:** 10.1186/s12879-023-08735-6

**Published:** 2023-11-02

**Authors:** Katherine Dai, Steffen Foerster, Neil M. Vora, Kathleen Blaney, Chris Keeley, Lisa Hendricks, Jay K. Varma, Theodore Long, Jeffrey Shaman, Sen Pei

**Affiliations:** 1 Department of Environmental Health Sciences, Mailman School of Public Health, Columbia University, 722 West 168th St, New York, NY 10032 USA; 2 Nuffield Department of Medicine, University of Oxford, Oxford, UK; 3 New York City Department of Health and Mental Hygiene (DOHMH), Long Island City, NY 11001 USA; 4 Department of Population Health Sciences, Weill Cornell Medical College, New York, NY 10065 USA; 5 NYC Health + Hospitals, New York, NY USA; 6 Department of Population Health, New York University, New York, NY 10016 USA; 7 Columbia Climate School, Columbia University, New York, NY 10025 USA

**Keywords:** Contact tracing, SARS-CoV-2, Delta variant, Transmission networks, Community transmission

## Abstract

**Background:**

Understanding community transmission of SARS-CoV-2 variants of concern (VOCs) is critical for disease control in the post pandemic era. The Delta variant (B.1.617.2) emerged in late 2020 and became the dominant VOC globally in the summer of 2021. While the epidemiological features of the Delta variant have been extensively studied, how those characteristics shaped community transmission in urban settings remains poorly understood.

**Methods:**

Using high-resolution contact tracing data and testing records, we analyze the transmission of SARS-CoV-2 during the Delta wave within New York City (NYC) from May 2021 to October 2021. We reconstruct transmission networks at the individual level and across 177 ZIP code areas, examine network structure and spatial spread patterns, and use statistical analysis to estimate the effects of factors associated with COVID-19 spread.

**Results:**

We find considerable individual variations in reported contacts and secondary infections, consistent with the pre-Delta period. Compared with earlier waves, Delta-period has more frequent long-range transmission events across ZIP codes. Using socioeconomic, mobility and COVID-19 surveillance data at the ZIP code level, we find that a larger number of cumulative cases in a ZIP code area is associated with reduced within- and cross-ZIP code transmission and the number of visitors to each ZIP code is positively associated with the number of non-household infections identified through contact tracing and testing.

**Conclusions:**

The Delta variant produced greater long-range spatial transmission across NYC ZIP code areas, likely caused by its increased transmissibility and elevated human mobility during the study period. Our findings highlight the potential role of population immunity in reducing transmission of VOCs. Quantifying variability of immunity is critical for identifying subpopulations susceptible to future VOCs. In addition, non-pharmaceutical interventions limiting human mobility likely reduced SARS-CoV-2 spread over successive pandemic waves and should be encouraged for reducing transmission of future VOCs.

**Supplementary Information:**

The online version contains supplementary material available at 10.1186/s12879-023-08735-6.

## Background

Since September 2020, the global circulation of SARS-CoV-2 has been characterized by the continual evolution of the novel coronavirus and the emergence of multiple new variants [1, 2]. The Delta variant (B.1.617.2), which emerged in India during late 2020, was first characterized in early 2021 [3]. It was designated a variant of concern (VOC) by the World Health Organization (WHO) on May 1, 2021. In the following months, the Delta variant rapidly spread across the world and replaced previously circulating VOCs [4].

Epidemiological studies have highlighted that the Delta variant was more transmissible than previous VOCs [5–7]. Studies from UK [8], Canada [9], Singapore [10] and Scotland [11] reported an increased risk of hospitalization for the Delta variant compared to the Alpha variant. Further studies suggested a substantial level of immunity escape for the Delta variant [12–15], resulting in re-infections among previously infected populations and breakthrough infections among vaccinated people [16–19].

Despite an improved understanding of the epidemiological features of the Delta variant, much remains unknown regarding how these characteristics manifest in population-level community transmission in urban settings. Addressing this knowledge gap is particularly challenging due to heterogeneous immunity during the Delta wave, caused by individual differences in natural infection and vaccination, as well as non-pharmaceutical interventions implemented to limit viral transmission. Data collected through contact tracing can provide valuable insights into the community transmission of infectious diseases [20]. In this study, we used large-scale contact tracing data in New York City (NYC) to understand community transmission of SARS-CoV-2 during the Delta wave. High-resolution data on close contacts (excluding facility investigation) and individual-level COVID-19 testing results linked to the contact tracing records enabled reconstruction of the transmission networks at individual and ZIP code levels.

## Methods

### Data sources

We analyzed the contact tracing data in NYC collected by the contact tracing team from May 11, 2021 to October 14, 2021. The contact tracing data contain information gathered from contact tracing phone calls, such as the age and home locations of index cases, their close contacts, and contact type. Contacts identified via facility investigations were excluded from the analysis. Index cases and their contacts were identified in the dataset using a matching algorithm based on personal identifying information (see details in Supplementary information). We cross-linked the contact tracing data with COVID-19 testing records provided by the NYC Department of Health and Mental Hygiene (DOHMH). Testing results for reported contacts were identified for the analysis. We refer to the contact tracing data and the cross-linked COVID-19 testing results as the Test and Trace dataset.

Demographic and socioeconomic data for NYC ZIP code tabulation areas (ZCTA) were compiled from the 5-year American Community Survey (ACS) [21]. COVID-19 surveillance data in NYC at the MOZCTA (modified ZIP code tabulation area) level are available at the GitHub repository maintained by DOHMH [22]. Vaccination data were obtained from the public repository of DOHMH [23]. Human mobility data recording the weekly number of visitors to points of interest (POIs, e.g., restaurants, grocery stores, etc.) in NYC were provided by SafeGraph [24], which aggregates anonymized location data from mobile phone applications to provide insights about physical places. We aggregated the mobility data to ZIP code level to estimate the weekly number of visitors (regardless of visitors’ location of residence) to POIs in each ZIP code area. We mapped the data from the ZCTA level to the MOZCTA level to align the geographical scale in the statistical analysis. In total, we collected data for all 177 MOZCTAs in NYC. For simplicity, we refer to MOZCTAs as ZIP code areas in the following analyses.

### Reconstructing transmission networks

Persons infected with SARS-CoV-2 can be contagious before symptom onset or even without symptoms [25–27]. Therefore, transmission might occur from an index case to a reported contact or vice versa. To reflect the uncertainty in the direction of transmission, we used a maximum-likelihood method to infer transmission chains based on the risk of transmission across different age groups. This method has been previously used to reconstruct transmission chains in the pre-Delta period [20]. Specifically, for symptomatic infections, we randomly sampled the infection date of each infected person using the earliest symptom onset date informed by the distribution of incubation period (i.e., interval from infection to symptom onset) [28]; for asymptomatic infections, we sampled the infection date using specimen collection date, where the interval from infection date to date testing positive was estimated using viral dynamics [29]. The sampled infection dates were used to determine the transmission direction between index cases and their infected contacts. We then used the Test and Trace data to estimate the probability of transmission for exposures across age groups. Finally, we randomly sampled 1000 possible transmission networks comprised of all inferred transmission links and selected the network that maximized the transmission likelihood using the cross-age group transmission probability. Further technical details can be found in Supplementary information.

To uncover potentially stable structural features of the transmission networks, we focused on a larger spatial scale than ZIP codes and identified communities, clusters of ZIP codes that were tightly connected by transmission. This approach acknowledges that the transmission of COVID-19 is not confined to neighboring ZIP codes and the patterns of connectivity between geographically distant communities may be persistent. We detected communities using a greedy search algorithm, which was chosen to optimize the modularity score [30, 31]. Modularity measures the strength of dividing a network into clusters: a network with high modularity has dense connections among the nodes within clusters but sparse connections between nodes in different clusters. This analysis was performed using the function “cluster_fastgreedy” in R package igraph [32].

### Statistical analysis

We used conditional autoregressive (CAR) models [33–35] to assess the associations of non-household within- and cross-ZIP code transmission with demographic, socioeconomic, disease surveillance, vaccination coverage, and human mobility features. Pearson correlation coefficients between these variables are shown in Fig. S1. Specifically, we fitted a Poisson generalized linear mixed model (GLMM) where the random effect was modeled by CAR priors to account for the inherent spatial-temporal autocorrelation present in the disease transmission data. Denote ${y}_{within}(i,t)$ as the weekly numbers of non-household within-ZIP code transmission events in ZIP code $i$ and week $t$. The model for ${y}_{within}(i,t)$ is described by:

1 $${log}\left({y}_{within}(i,t)\right)={ log}\left(population\left(i\right)\right)+{\beta }_{1}\times {log}\left(population\; density\left(i\right)\right)+{\beta }_{2}\times \text{log}\left(weekly\;cases\;per\;capita\left(i,t\right)\right)+{\beta }_{3}\times \text{log}\left(weekly\;tests \;per\;capita\left(i,t\right)\right)+{\beta }_{4}\times cumulative\;cases\;per\;capita\left(i,t\right)+{\beta }_{5}\times \%\;Black\;resident\left(i\right)+{\beta }_{6}\times \%\;Hispanic\;resident\left(i\right)+{\beta }_{7}\times \%\;resident\;over\;65\left(i\right)+{\beta }_{8}\times median\;household\;income\left(i\right)+{\beta }_{10}\times mean\;household\;size\left(i\right)+{\beta }_{11}\times \%\;fully\;vaccinated\;resident\left(i, t\right)+{\beta }_{12}\times weekly\;POI\;visitors\;per\;capita\left(i,t\right)+{\psi }_{it}+{\epsilon }_{it}.$$

Here ${ log}\left(population\left(i\right)\right)$ is the offset, ${\psi }_{it}$ is the random effect for location $i$ and week $t$, and ${\epsilon }_{it}$ is the error term. All covariates were standardized (mean zero and standard deviation one) before the regression analysis. The unit of each variable (i.e., the standard deviation of the original data) is reported in Table S1. We used log-transformed population as an offset, assuming the numbers of transmission events are proportional to local population. We used weekly cases per capita to represent the local force of infection that impacts the number of observed transmission events. The model for cross-zip code transmission is defined using the same Eq. (1). In the final model, we aimed to minimize multicollinearity by ensuring that the variance inflation factor (VIF) was less than 5 for all variables. While additional variables, such as percent bachelor’s and the opportunity to work from home, are expected to influence transmission, both were found to be correlated with the existing variables in the model. The models were implemented using the R package CARBayesST, in which model coefficients and parameters were estimated using a Markov chain Monte Carlo (MCMC) algorithm. Details on the implementation of the MCMC algorithm can be found in Ref. [33].

## Results

**Fig. 1:**
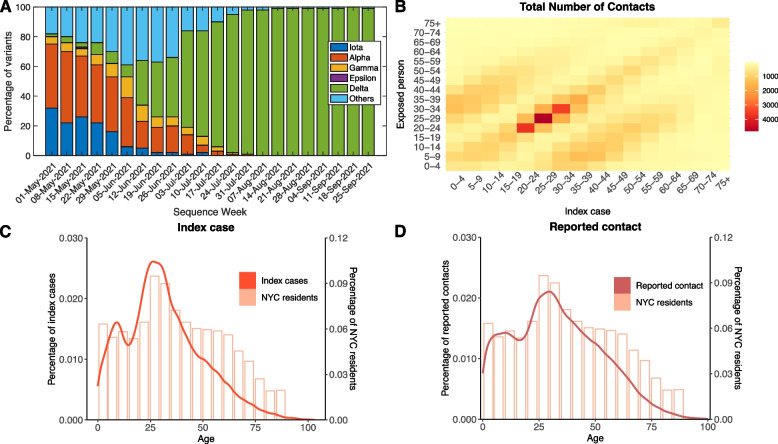
Emergence of the Delta variant and contact patterns in NYC. **A** Percentage of SARS-CoV-2 variants in NYC from May 1 2021 to September 25 2021. The ancestral virus strain is classified as part of “Others”. Data were obtained from the NYC DOHMH public repository (https://github.com/nychealth/coronavirus-data/tree/master/variants). **B** The contact mixing matrix showing the total number of reported contacts among age groups during the study period. **C**, **D** Age distributions of index cases and self-reported contacts (solid lines). The age distribution of NYC residents is shown by pink bars

### Population mixing and age profile of Infection

During the study period, the Delta variant displaced other VOCs and became dominant in the city (Fig. 1A) [22]. We observed a typical three-band pattern in the contact matrix across age groups, representing frequent contacts among people with similar ages and inter-generational contacts within household (Fig. 1B). In particular, the number of reported contacts among young adults (20 to 40 years old) was considerably higher than other age groups. Young adults constituted the majority of index cases, similar to the pre-Delta period [20]; however, an increased number of children and adolescents below 15 years old were infected during the Delta wave (Fig. 1C). Young adults aged 20 to 40 years old were also more likely to be reported as close contacts (Fig. 1D). Compared with the pre-Delta period, infections among children and adolescents became more prevalent.

**Fig. 2:**
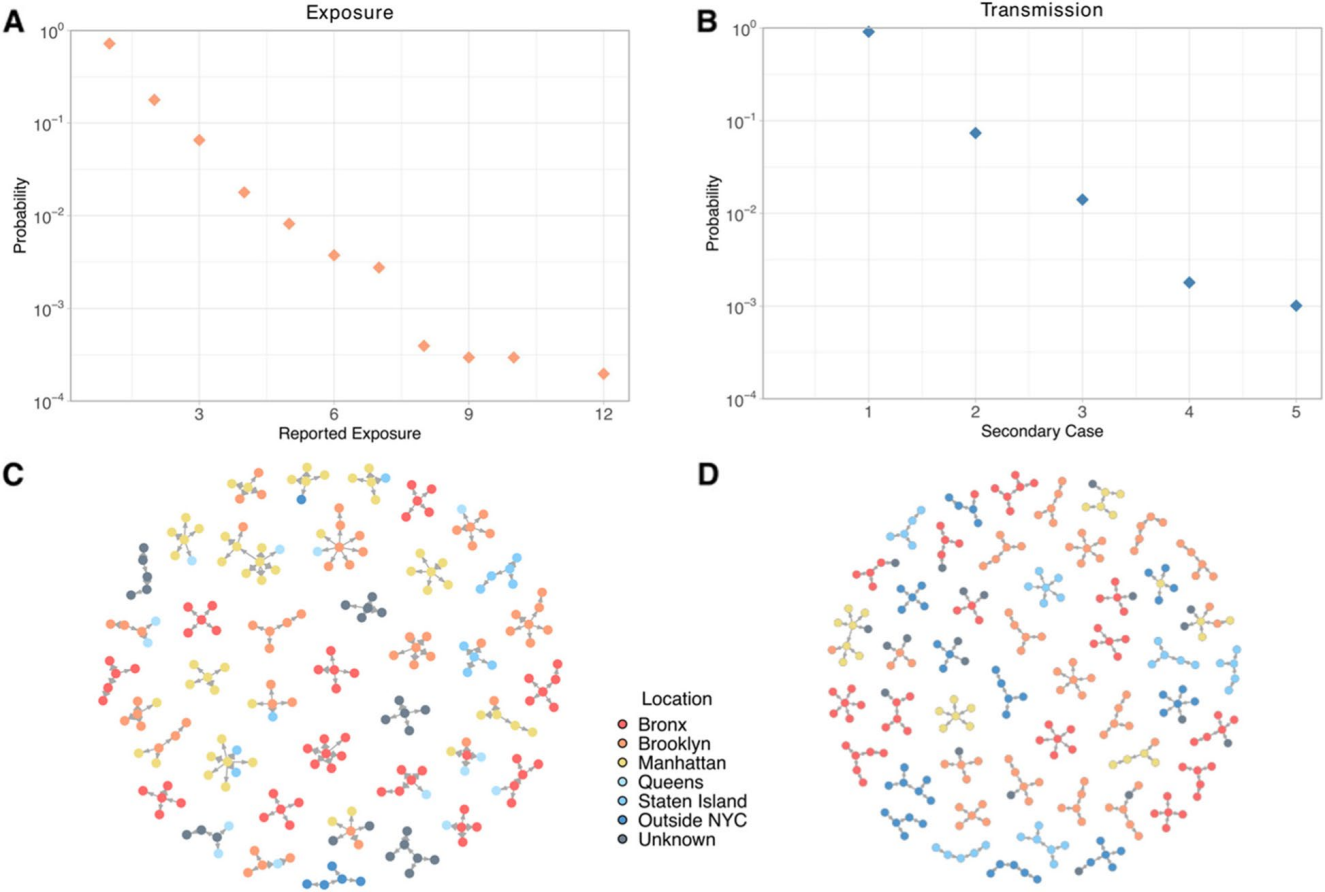
Structure of exposure and transmission networks. **A** Distribution of the number of reported contacts. **B** Distribution of secondary cases caused by index cases identified in the testing results. **C**–**D** Examples of exposure and transmission networks. Colors indicate the home location of each person (five boroughs in NYC, outside NYC, and unknown)

### Structure of exposure and transmission networks

We reconstructed the exposure networks between index cases (both persons tested positive and symptomatic contacts who completed case investigation) and contacts. The number of contacts reported by cases was highly skewed (Fig. 2A), with most index cases reporting less than three close contacts. We further reconstructed the transmission networks between index cases and their contacts who tested positive using PCR or antigen tests. Here we excluded probable cases in contacts from the transmission network and focused on confirmed transmission events. The number of confirmed secondary infections per index case had a large individual variation (Fig. 2B). We visualized examples of exposure networks (Fig. 2C) and transmission networks (Fig. 2D), using colors to indicate the borough where each person lives (Bronx, Brooklyn, Manhattan, Queens, Staten Island, outside NYC, and unknown). While most exposures and transmission events involved residents living within the same borough, cross-borough exposures and transmissions were also observed. The heterogeneity of exposure and transmission networks was comparable to that of the pre-Delta period [20, 28, 36, 37] and therefore might be a persistent epidemiological feature of SARS-CoV-2. However, the maximum numbers of reported contacts and secondary infections were lower (exposure: pre-Delta 77 versus Delta 12; infection: pre-Delta 7 versus Delta 5).

**Fig. 3:**
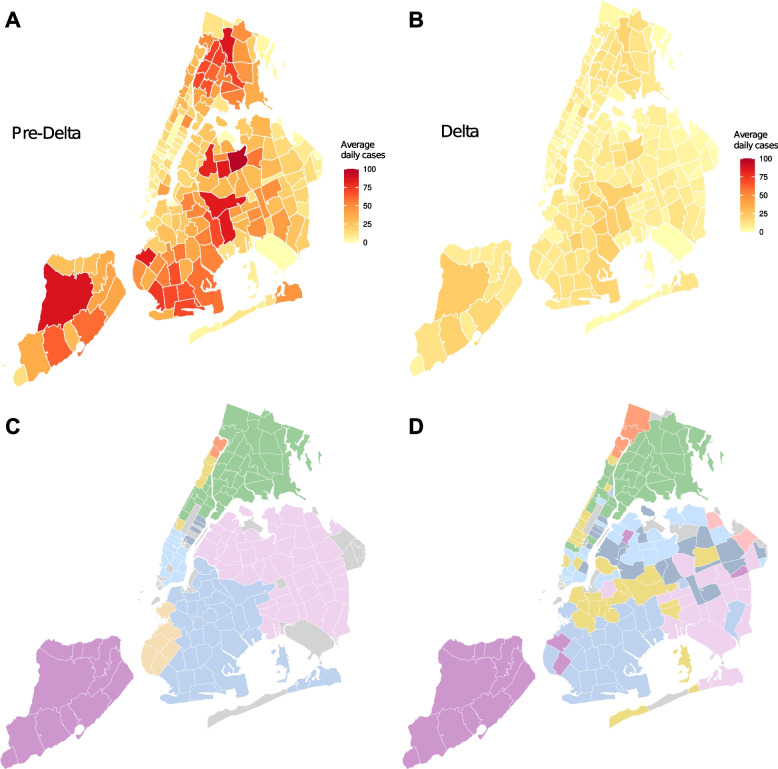
Community transmission of SARS-CoV-2 during the second wave and the Delta wave in NYC. **A** The cumulative number of reported COVID-19 cases in each ZIP code from October 1 2020 to May 10 2021. **B** The cumulative number of reported COVID-19 in each ZIP code from May 11 2021 to October 14 2021. **C**, **D** Clusters of ZIP code areas that were strongly connected by transmission events during the second wave (**C**) and the Delta wave (**D**). Clusters were identified using a network community detection method applied to weighted networks. Different clusters are highlighted using colors

### Community transmission of SARS-CoV-2

We compared cumulative reported cases at the ZIP code level in the pre-Delta and Delta periods (Fig. 3A-B). Staten Island and the Bronx reported a large number of infections during the Delta wave (Fig. 3B), similar to the pattern in the pre-Delta period (Fig. 3A). The number of confirmed cases in Manhattan remained low before and after the emergence of Delta. In contrast, certain communities in Queens and Brooklyn with high transmission during the pre-Delta period were less impacted by the Delta variant.

To examine the spatial transmission pattern across ZIP code areas, we aggregated cross-ZIP code transmission events to form a weighted network with directed links. Each node represents one ZIP code area, and the weight of each directed link represents the number of transmission events from one location to another. We used network community detection to identify clusters of ZIP codes that were tightly connected by transmission within each cluster. The resulting network communities, highlighted with distinct colors, were compared for the pre-Delta period (Fig. 3C) and the Delta wave (Fig. 3D). Some ZIP code clusters in locations such as the Bronx, Staten Island and southern Brooklyn persisted over time; however, ZIP code clusters during the Delta wave were more fragmented in Queens, Brooklyn, and Manhattan, indicating a greater number of small clusters with strong localized transmission. In addition, we found that the number of weekly visitors increased during the Delta wave, compared to the pre-Delta period (*p* < 0.001). Given the higher transmissibility of the Delta variant and increased mobility during the summer of 2021, both local and long-range transmission occurred more frequently. The increase in long-range transmission, in particular, may have created non-local transmission clusters. We examined distributions of index cases who initiated transmission and their infected contacts across ZIP codes and found that certain ZIP codes were more involved in the spatial spread of COVID-19 (Fig. S2). Geographically, most cross-ZIP code transmission events occurred within 10 km; however, longer-distance transmission was also evident (Fig. S2).

**Fig. 4:**
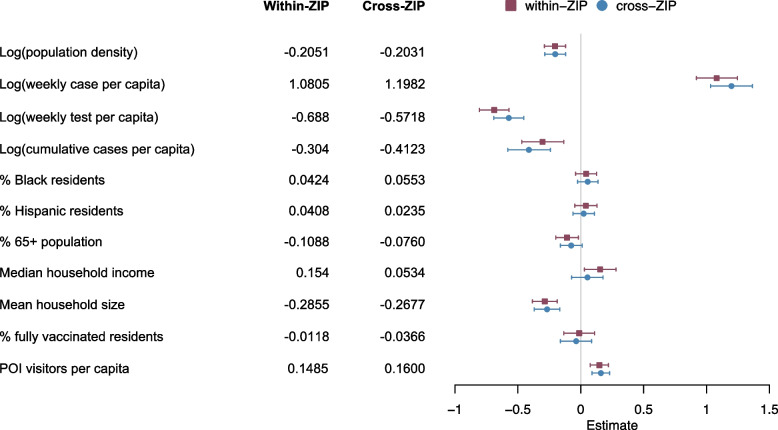
Effects of various features on the transmission of SARS-CoV-2 in NYC during the Delta wave. Coefficients for non-household within-ZIP code transmission and cross-ZIP code transmission are shown for 11 covariates. Median estimates and 95% CIs are presented. The unit of each variable is reported in Table S1

### Factors associated with COVID-19 spread

For both within- and cross-ZIP code transmission, the model identifies strong negative effects of population density, weekly tests per capita, and cumulative cases per capita, as well as a strong positive effect of weekly cases per capita (Fig. 4). Thus, during the Delta wave, ZIP code areas with lower population density, a higher ongoing infection, a lower testing effort, and a lower level of prior infection (measured by cumulative confirmed cases in each location) had more transmission events. A larger mean household size is associated with lower transmission for both within- and cross-ZIP code spread. Vaccination was not found to be associated with SARS-CoV-2 transmission during the Delta wave, while increased POI visitors per capita is associated with a higher level of within- and cross-ZIP transmission. The model did not find a significant association between transmission and most demographic (% Black residents, % Hispanic residents, % 65 + population) and socioeconomic (Median household income, % residents with bachelor) factors (except for a weak effect of % 65 + population on cross-ZIP code transmission).

### Discussion and conclusions

In this study, using detailed contact tracing data and testing records, we analyzed the transmission of SARS-CoV-2 during the Delta wave in NYC from May 2021 to October 2021. During the study period, the transmission of SARS-CoV-2 is likely driven by young adults aged 20 to 40 years old, consistent with the pre-Delta period. However, infections among children and adolescents below 15 years old substantially increased. Potential causes include the low vaccination coverage in this subgroup (vaccines for adolescents aged 12 through 15 became available in May 2021) and increased transmissibility of the Delta variant. Heterogeneity in the numbers of close contacts and secondary infections persisted during the Delta wave, but the maximum number for self-reported contacts was lower.

We observed a less clustered community transmission of SARS-CoV-2 among ZCTAs during the Delta wave, with more frequent long-range transmission events occurring in Queens, Brooklyn, and Manhattan. This is likely the result of the increased transmissibility of the Delta variant and increased human contact following the gradual relaxation of control measures. Coupled with the increase in local transmission, the increase in long-range transmission of SARS-CoV-2 across NYC communities made containment of the virus in the metropolitan area challenging.

Many of the findings are at odds with the results from the same analysis for the pre-Delta period [20]. For instance, the effects of population density and mean household size have opposite signs, and the effects of many factors that were previously found significant disappeared during the Delta wave. These varied findings are possibly due to changes to population immunity in different ZIP code areas acquired from prior infection. In general, communities hit hard in the pre-Delta period (with high population density and large household size) may have possessed higher immunity against the Delta variant. Prior infection can confer protection against repeat infection with the Delta variant, modulating transmission of subsequent VOCs. The effect of vaccination on population-level transmission was not observed, possibly due to high overall vaccine coverage across the city. Our findings underscore the importance of documenting variability in immunity across a population, i.e. the ‘immunity landscape’, to identify sub-populations at potential high-risk for infection by future VOCs [38]. With a complex combination of natural infection, vaccination, boosting, re-infection and breakthrough infection, the evolution and transmission of new variants will be shaped by this immunity landscape. POI visitors per capita were found to be a significant driver of SARS-CoV-2 transmission, as during prior waves. This implies that non-pharmaceutical interventions limiting person-to-person contact likely remained effective over successive pandemic waves and are a viable option for reducing transmission of VOCs.

A few limitations exist in this study. Firstly, self-reported contact tracing data are subject to observational biases. These biases include differential reporting rates by contact type (overwhelmingly biased to household contacts) and age group, and the tendency of contacts to get tested. Due to the incomplete records of contacts as well as strong under-reporting of infections [39, 40], identified exposure and transmission networks are highly fragmented. As a result, the transmission networks are more reflective of ego-networks given the extremely incomplete elicitation of contacts and missed transmissions. This likely has implications for generalizing the findings of the observed network structure to the population level. Secondly, several VOCs co-circulated in May and June of 2021. Findings in this study are not exclusively for the Delta variant. Thirdly, matching of close contacts and their testing results may be incomplete due to missing and incorrect personal identifying information. Lastly, human mobility data derived from mobile devices may have been biased across POI types and age groups.

### Supplementary Information

**Additional file 1.**

## Data Availability

COVID-19 surveillance data in NYC at the MOZCTA (modified ZIP code tabulation area) level are publicly available at the GitHub repository maintained by the NYC Department of Health and Mental Hygiene (DOHMH) (https://github.com/nychealth/coronavirus-data). Demographic and socioeconomic data for NYC zip code tabulation areas (ZCTA) are available from the 5-year American Community Survey (ACS) (https://www.census.gov/programs-surveys/acs/data.html). Contact tracing records and individual testing results are subject to restrictions for the protection of patient privacy. Requests for data access should be addressed to NYC DOHMH and NYC Health + Hospitals or the corresponding author. The corresponding author will respond to requests within two weeks and facilitate communications with NYC DOHMH and NYC Health + Hospitals, who will provide details of any restrictions imposed on data use via data use agreements.
