# Supplementary material for: Community transmission of SARS-CoV-2 during the Delta wave in New York City
Source: BMC Infect Dis. 2023 Nov 2;23:753. doi: 10.1186/s12879-023-08735-6 (PMC10621074; doi:10.1186/s12879-023-08735-6)
Supplement: Supplementary file 1 — Additional file 1. [file 12879_2023_8735_MOESM1_ESM.docx]

**Supplementary Information**

**1. Cross-link contact tracing dataset and individual COVID-19 testing results**

We used a two-step matching process to match individuals between the contact tracing and DOHMH surveillance databases. In the first, heuristic step, pairwise matching was performed with blocking on any phone number and date of birth, requiring either of the two fields to be non-missing and both records to match exactly. Case-insensitive optimal string alignment distance was then computed between first names and last names of pairwise entries of blocked records to detect minor misspellings. Diminutive first names were flagged using text searching. String distance thresholds of two or less for the last name and one or zero or a diminutive flag for the first name were used to identify positive matches. If matched pairs from this heuristic matching process had non-matching person identifiers from the DOHMH and T2 databases or person identifiers were missing for at least one of the paired records, records entered a second matching step. For this step, an XGBoost model was trained using the following features: 1) Jaro-Winkler string distance score for first and last name; 2) optimal string alignment distance between phone numbers, allowing for transposition between home and mobile phones; when missing, 10 was imputed; 3) the count of month/date/year components of the dates of birth that matched exactly, accounting for the possibility of components being mis-entered into the database; when missing, 3 was used; 4) a flag to identify the transposition of month and date components of the dates of birth; 5) the difference in birth years as an absolute value; when missing, a difference of 90 was imputed; 6) the probability distribution of the first and last name; 7) optimal string alignment distance between normalized mailing addresses and zip codes; when missing, they were assumed to be different and a distance of 40 was imputed for mailing address and 5 for zip code. All predictors were standardized to z-scores. A gold-standard data set was created by subjectively hand-labeling a random sample of 2,886 step 2 input matches as good/bad matches based on the personally identifying information. The XGBoost model was trained and tuned using five-fold cross validation on an 80/20 training/test split, with stratification on the binary (good/bad match) target variable. The Area Under the Curve (AUC) was maximized at 0.937 with the following hyperparameters: nrounds = 10, max_depth = 6 and eta=0.3. We reviewed sensitivity, specificity and F1 scores on the test dataset at different predicted probability cut-off values to identify the threshold above which record pairs were automatically matched. We selected a predicted probability of a match of 0.5 as the final threshold; this choice was based on a near-maximum F1 score at this threshold, and because it is widely used in machine learning applications as the threshold of choice. The sensitivity at this cut-off was 0.957, specificity was 0.702 and precision was 0.922. Step 2 input matches that were classified as “good” matches moved on to the final matches and combined with step 1 matches. Final matches were joined to each other to turn a = b and b = c pairs into a = b = c relationships and assigned person IDs. Records that did not make it to the final matches list were treated as people represented by only one record and were each assigned a unique person ID.

**2. Reconstructing transmission networks**

Due to asymptomatic and pre-symptomatic shedding, the reporting dates of index cases and contacts cannot be used to determine the direction of transmission. To address this issue, we developed a maximum-likelihood method to reconstruct transmission chains based on the risk of COVID-19 spread across different age groups. This approach includes three steps:

- 1. Estimate the infection time using symptom onset date or specimen collection date. Use the estimated infection time to determine the direction of exposure and transmission.
  2. Estimate the probability of transmission for exposures across age groups using test and trace data.
  3. Sample an ensemble of possible transmission networks and select the one that maximizes the transmission likelihood.

**2.1 Estimation of infection time**

For each pair of index case and contact, we inferred the direction of exposure or transmission using estimated infection time. All index cases were confirmed infections, but only a proportion of contacts were tested. We therefore used exposure pairs for which both the index case and contact had been tested and excluded exposure pairs for which contacts had not been tested, as these contacts did not affect the observed transmission network. If the contact tested negative, the direction of exposure is from the index case to the close contact (i.e., the index case is the infector); however, if the contact tested positive, the direction of exposure is uncertain and must be estimated.

For symptomatic cases who reported symptom onset dates, the infection time was estimated using the distribution of the incubation period reported from previous studies. Incubation period is the time between infection and symptom onset. Here we used a Weibull distribution estimated based on detailed contact tracing data from Hunan province, China^1^. Specifically, the probability density function (PDF) for the incubation distribution is

$$p\left( x \right)=\frac{k}{\lambda}\left( \frac{x}{\lambda} \right)^{k-1}e^{-\left( \frac{x}{\lambda} \right)^{k}}, [1]$$

where the shape parameter $k=1.58$ and the scale parameter $\lambda=7.11$. To estimate infection time, we randomly drew incubation periods (in days) for symptomatic cases from this PDF [1].

For cases without symptoms, we used specimen collection date to estimate infection date. Denote $t_{inf\to test}$ as the interval from infection to specimen collection date. We aim to estimate $t_{inf\to test}$ given a person tested positive, i.e., $P\left( t_{inf\to test} | positive \right)$. Using Bayes’ rule, we have the following relation:

$$P\left( t_{inf\to test} | positive \right)\propto P\left( t_{inf\to test} \right)P\left( positive | t_{inf\to test} \right). [2]$$

Here $P\left( t_{inf\to test} \right)$ is the prior and $P\left( positive | t_{inf\to test} \right)$ is the likelihood of testing positive given that specimens were collected $t_{inf\to test}$ days after infection.

The prior $P\left( t_{inf\to test} \right)$ can be approximated using the interval from infection to specimen collection date for symptomatic cases, which provides a roughly plausible range of $t_{inf\to test}$. For each tested symptomatic case, $t_{inf\to test}$ is the sum of the sampled incubation period (from infection to symptom onset) and the time from symptom onset to specimen collection date, available in the dataset.

The likelihood $P\left( positive | t_{inf\to test} \right)$ was estimated using viral dynamics and limits of detection (LOD) for PCR tests. Following Larremore et al.^2^, we generated synthetic viral dynamics in infected persons. The log-transformed viral load (copies/mL), $V$, is a piece-wise linear function of the number of days after infection, $t$. Specifically, the viral dynamics is determined by three control points: $(t_{0}, 3)$, $(t_{peak}, V_{peak})$, and $(t_{f}, 6)$. Here $t_{0}$ is the time when log viral load reaches 3; $t_{peak}$ is the peak timing of log viral load; $V_{peak}$ is the peak magnitude of log viral load; and $t_{f}$ is the time when log viral load falls to 6. The mean log viral load was computed using the following function:

$$\bar{V}(t)=\left\{ \begin{aligned} \frac{3t}{t_{0}}, t\leq t_{0} \\ 3+\frac{\left( V_{peak}-3 \right)\left( t-t_{0} \right)}{t_{peak}-t_{0}}, t_{0}<t\leq t_{peak} \\ \max\left( V_{peak}-\frac{\left( V_{peak}-6 \right)\left( t-t_{peak} \right)}{t_{f}-t_{peak}},0 \right), t>t_{peak} \end{aligned} \right. [3]$$

In simulations, the following parameter distributions were used: $t_{0}\sim U(2.5, 3.5)$, $V_{peak}\sim U[7,11]$, $t_{peak}\sim\min\left( t_{0}+0.5+\Gamma\left( 1.5,1 \right), 3 \right)$, and $t_{f}\sim t_{peak}+U(2,6)$. Here $U(a,b)$ is a uniform distribution between $a$ and $b$; and$\Gamma(a,b)$ is a Gamma distribution with a shape parameter $a$ and a scale parameter $b$. The simulated log viral load on each day $t$ was drawn from a Gaussian distribution:

$$V\left( t \right)\sim N\left( \mu=\bar{V}\left( t \right), \sigma^{2}=0.04\bar{V}\left( t \right)^{2} \right). [4]$$

We simulated 10^5^ viral load trajectories. For each trajectory, we randomly drew a LOD from $U[2,3.5]$, defined as the threshold for positive results – the test result is positive if $V(t)$ is above the LOD and negative otherwise. Using simulated viral dynamics and LOD, we obtained the likelihood $P\left( positive | t_{inf\to test} \right)$.

We computed the posterior distribution $P\left( t_{inf\to test} | positive \right)$ using Eq. [2]. The infection time for cases without symptoms was estimated using the distribution $P\left( t_{inf\to test} | positive \right)$. Once the infection time of index case - contact pairs had been sampled, using either symptom onset date or specimen collection date, the direction of exposure could be determined by the chronological order of infection.

**2.2 Estimation of transmission probability across age groups**

We further use the test and tracing date to estimate the transmission probability across age groups, which are used to reconstruct transmission chains. We classify the total population into four age groups: 0-9, 10-19, 20-64, and 65+. Denote $P_{a\to a^{'}}(positive)$ as the probability of successful transmission for an exposure from age group $a$ to $a'$. In actuality, we only observe $P_{a\to a^{'}}(positive|test)$ among tested exposures. Bayes’ rule gives

$$P_{a\to a^{'}}\left( positive \right)=P_{a\to a^{'}}\left( positive | test \right)\times\frac{P_{a\to a^{'}}\left( test \right)}{P_{a\to a^{'}}\left( test | positive \right)}, [5]$$

where $P_{a\to a^{'}}\left( test \right)$ is the probability that an exposure from age group $a$ to $a'$ is tested and $P_{a\to a^{'}}\left( test | positive \right)$ is the probability of testing given a successful transmission from age group $a$ to $a'$, i.e. an infection.

If we assume the relative test-seeking probability between exposed and infected individuals is independent of age, then $P_{a\to a^{'}}\left( test \right)/P_{a\to a^{'}}\left( test | positive \right)$ is constant across age groups. We then can use the test positivity rate for exposure from age group $a$ to $a'$, $P_{a\to a^{'}}\left( positive | test \right)$, to represent the relative transmission probability across age groups: $P_{a\to a^{'}}\left( positive \right)=\gamma P_{a\to a^{'}}\left( positive | test \right)$, where $\gamma=P_{a\to a^{'}}\left( test \right)/P_{a\to a^{'}}\left( test | positive \right)$.

To compute $P_{a\to a^{'}}(positive|test)$, we first used the method introduced in subsection 2.1 to determine the possible directions of exposure pairs for which both index case and contact were tested. Then we selected the pairs of exposures from age group $a$ to $a'$ and computed the probability of successful transmission for $a\to a^{'}$ exposures. We repeated this analysis 1,000 times and took the average transmission probability $P_{a\to a^{'}}(positive|test)$.

**2.3 Reconstruction of the maximum likelihood transmission network**

We combined the methods described in subsections 2.1 and 2.2 to reconstruct transmission networks. Using the method developed in subsection 2.1, we first estimated the possible directions of transmission events (in which both index case and contact tested positive) and used these directed transmission links to form a putative transmission network. For each transmission link $\mathcal{l}$, we identified the age groups for both patients (e.g., an exposure from age group $a$ to $a'$) and recorded the transmission probability across age groups for this link $\mathcal{l}$ (estimated in subsection 2. 2) as $P_{\mathcal{l}}\left( positive | test \right)$. We computed the likelihood considering all transmission links: $L=\sqrt[n]{\prod_{\mathcal{l}} P_{\mathcal{l}}(positive|test)}$ where $\mathcal{l}$ runs over all $n$ transmission links in the network. We sampled 1,000 putative transmission networks and selected the network that maximizes the likelihood $L$ among the ensemble of possible transmission networks.

**References**

1. Hu, S. *et al.* Infectivity, susceptibility, and risk factors associated with SARS-CoV-2 transmission under intensive contact tracing in Hunan, China. *Nat. Commun.* **12**, 1533 (2021).

2. Larremore, D. B. *et al.* Test sensitivity is secondary to frequency and turnaround time for COVID-19 screening. *Sci. Adv.* **7**, eabd5393 (2021).

Fig. S1. Pearson correlation coefficients between pairs of variables in the regression analysis. Correlation coefficients were computed after data standardization.


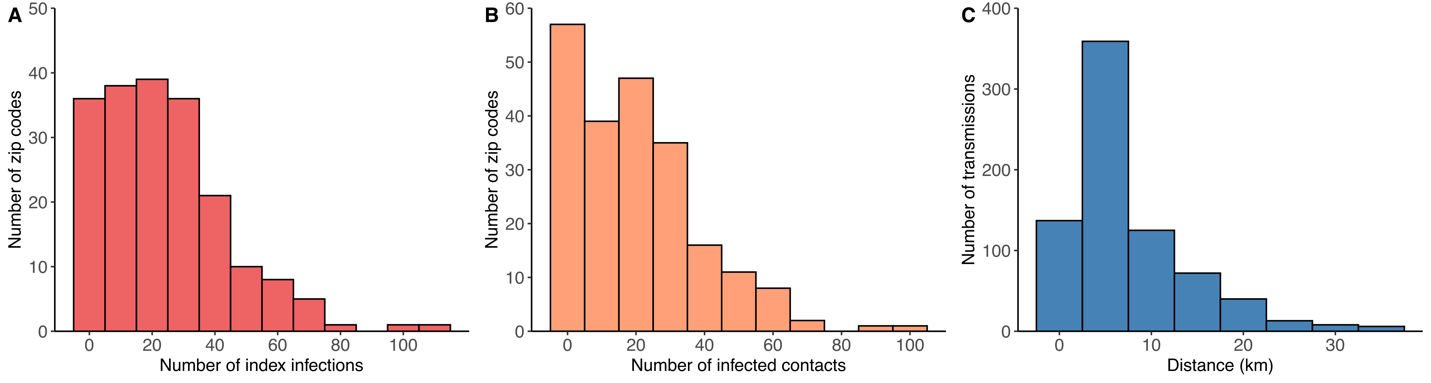


Fig. S2. Cross-ZIP code transmission in NYC. The distributions of index infections and infected contacts across ZIP code areas are presented in A and B. C shows the distribution of distance between home ZIP codes of index infections and infected contacts in cross-ZIP code transmission events. The population weighted centroids for ZIP code areas were used to compute the distance.

Table S1. Units of variables included in statistical analysis. Units were computed as standard deviations of variables before standardization.

| Variable | Standard deviation |  |
| --- | --- | --- |
| Population density | 31860.43 | People per square kilometer |
| Weekly case per capita | 76.78 | Cases per 100,000 people |
| Weekly test per capita | 1481.42 | Tests per 100,000 people |
| Cumulative cases per capita | 672.45 | Cumulative cases per 100,000 people |
| % Black residents | 23% | Percent |
| % Hispanic residents | 19% | Percent |
| Median household income | 38635.59 | Dollars |
| % 65+ population | 5% | Percent |
| Household size | 0.48 | People per household |
| % fully vaccinated residents | 13% | Percent |
